# Innate immune response after acute myocardial infarction and pharmacomodulatory action of tacrolimus in reducing infarct size and preserving myocardial integrity

**DOI:** 10.1186/1423-0127-20-82

**Published:** 2013-10-29

**Authors:** Jiunn-Jye Sheu, Pei-Hsun Sung, Steve Leu, Han-Tan Chai, Yen-Yi Zhen, Yi-Ching Chen, Sarah Chua, Yung-Lung Chen, Tzu-Hsien Tsai, Fan-Yen Lee, Hsueh-Wen Chang, Sheung-Fat Ko, Hon-Kan Yip

**Affiliations:** 1 Division of Thoracic and Cardiovascular Surgery, Department of Surgery, Kaohsiung Chang Gung Memorial Hospital and Chang Gung University College of Medicine, Kaohsiung, Taiwan; 2 Division of Cardiology, Department of Internal Medicine, Kaohsiung Chang Gung Memorial Hospital and Chang Gung University College of Medicine, Kaohsiung, Taiwan; 3 Center for Translational Research in Biomedical Sciences, Kaohsiung Chang Gung Memorial Hospital and Chang Gung University College of Medicine, Kaohsiung, Taiwan; 4 Department of Biological Sciences, National Sun Yat-Sen University, Kaohsiung, Taiwan; 5 Department of Radiology, Kaohsiung Chang Gung Memorial Hospital and Chang Gung University College of Medicine, Kaohsiung, Taiwan

## Abstract

**Background:**

This study investigated the association between innate immune reaction and myocardial damage after acute myocardial infarction (AMI) and anti-inflammatory role of tacrolimus in reducing infarct size. Male mini-pigs (n=18) were equally categorized into sham control (SC), untreated AMI (by ligation of left anterior descending coronary artery), and AMI-Tacrolimus (AMI-Tac) (0.5 mg intra-coronary injection 30 minutes post-AMI). Cardiac magnetic resonance imaging (MRI) was performed at post-AMI days 2, 5 and 21 before sacrificing the animals.

**Results:**

By post-AMI day 21, left ventricular ejection fraction (LVEF) was lowest in untreated AMI animals, significantly higher in SC than in AMI-Tac group (all p<0.003). Infarct areas at basal, middle, and apical levels, numbers of CD14+ and iNOS+ cells in infarct area (IA) and peri-IA, and protein expression of CD14, CD68, and Ly6g from circulating inflammatory cells showed an opposite pattern compared with that of LVEF in all groups (all p<0.005). Protein expressions of MCP-1, MIP-1, TNF-α, NF-κB, iNOS, and IL-12 in IA and peri-IA exhibited an identical pattern compared to that of CD14, CD68, and Ly6g from circulating inflammatory cells (all p<0.01). Expressions of myocardial damage biomarkers in IA and peri-IA [γ-H2AX, β-myosin heavy chain (MHC), Smad3, TGF-β] were highest in AMI and higher in AMI-Tac than in SC, whereas expressions of myocardial integrity biomarkers (connexin43, mitochondrial cytochrome-C, α-MHC, BMP-2, Smad1/5) were opposite to those of damage biomarkers (all p<0.001).

**Conclusion:**

Innate immune responses were markedly augmented and LVEF was significantly reduced after AMI but were remarkably improved after tacrolimus treatment.

## Background

Acute myocardial infarction (AMI) is the leading cause of death worldwide [1-3]. The principal reason for poor clinical outcomes after AMI is mainly due to left ventricular (LV) remodeling following AMI that results in LV dilatation and pump failure [4-7]. Since the essential etiology of pump failure is closely related to the severity of myocardial damage and necrosis after AMI [4-9], the first priority in the management of AMI is effective alleviation of myocardial damage and salvage of jeopardized myocardium after AMI.

Despite prompt reperfusion [i.e., primary percutaneous coronary intervention (PCI) or thrombolytic therapy] is the current golden standard in the treatment of AMI [10-12], unsuccessful reperfusion, including failure in resuming patency of the infarct artery through thrombolysis or slow-flow/no reflow after primary PCI [12-14], remains the Achilles’ heel leading to unfavorable clinical outcome [10,12,13]. The underlying mechanisms involved in slow or no-reflow phenomenon have been extensively investigated [12,14,15]. Previous reports have shown that high-burden thrombosis formation and plaque content as well as ischemia-reperfusion injury (IRI) are the principal causes of unsuccessful reperfusion [12,14-17]. Additionally, IRI has been further demonstrated to play the key role for propagation of myocardium damage even after successful reperfusion. Moreover, the phenomenon of IRI has also been identified to be essential for up-regulating inflammatory response [14,18-20]. Of particular importance is that the role of inflammation has been highlighted by numerous recent studies as a critical contributor to unsuccessful reperfusion, myocardial damage and death after AMI [19,21-25]. Therefore, early inhibition of propagation of inflammatory reaction other than reperfusion therapy may be also an important therapeutic strategy against progression of AMI-induced myocardial damage which in turn, causes the LV dysfunction and remodeling.

It is well-known that innate and adaptive immune systems are the two major defenses of the host against micro-bacterial, viral, or other antigenic stimulations. In contrast to the adaptive immune system that would need time to act against the antigen (i.e., from memory to immune cell differentiation and maturation), the non-specific innate immune reaction always take quickly response to the inflammatory or antigen-related stimulation. Since AMI elicits prompt tissue necrosis and inflammation, it is conceivable that innate immunity has an important role to play a rapidly immune-inflammatory response, especially at the early phase of AMI. Surprisingly, while the link between AMI and inflammatory reaction has been investigated in depth [21-27], the involvement of the innate immune system in inflammation after AMI has only been seldom reported [25,27].

Calcineurin, which possesses serine/threonine phosphatase activity, binds to nuclear factor of activated T cells (NF-AT) which is then dephosphorylated and translocated to the nucleus for activating gene expression of cytokines [28]. Tacrolimus, also named FK506, is a macrolide immunosuppressant acting via lymphokine signal transduction pathway [29]. Tacrolimus forms the FK506-binding protein (FK-BP) (i.e., immunophilin FKBP12) in cytoplasmic receptor which inactivates calcineurin which is a central phosphatase for T cell signaling [29,30]. Our recent experimental studies [26,27] have revealed that tacrolimus treatment effectively attenuated inflammation and MAPK signaling, limited infarct size, and preserved LV function. However, our study did not further investigate the potentially important role of innate immunity after AMI, and the LV ejection fraction (LVEF) was only measured by M-mode echocardiography that would under- or over-estimate heart function. Accordingly, the present study, using a mini-pig AMI model, attempted to assess AMI-elicited activations of innate immune signaling and particularly to determine the role of tacrolimus in inhibiting inflammation and preserving myocardial integrity as well as to evaluate LV function after AMI using a more accurate tool of cardiac magnetic resonance imaging (MRI).

## Methods

### Ethics

All animal experimental procedures were approved by the Institute of Animal Care and Use Committee at Chang Gung Memorial Hospital – Kaohsiung Medical Center (Affidavit of Approval of Animal Use Protocol No. 2008121108) and performed in accordance with the Guide for the Care and Use of Laboratory Animals (NIH publication No. 85–23, National Academy Press, Washington, DC, USA, revised 1996). The IRB number of this animal study was 2011070502.

### Mini-Pig AMI model

The procedure and protocol of the animal model of AMI and the rationale of drug dosage were based on our recent report [26]. Briefly, each male mini-pig (Taitung Animal Propagation Station, Livestock Research Institute, Taiwan), weighing 16–18 kg, was anesthetized by intramuscular injection of ketamine (15 mg/kg) and maintained in anesthetized condition using an inhalation of 1.5% isoflurane for the whole procedure. After being shaved on the chest, the mini-pig was placed in supine position on a warming pad at 37°C and then received endotracheal intubation with positive-pressure ventilation (180 mL/min) with room air using a ventilator (Sn: Q422ZO, SIMS PneuPAC, Ltd.) during the procedure. Electrocardiogram (ECG) monitor and defibrillator were connected to the chest wall of each mini-pig. One ampoule of amiodarone (150 mg) was intravenously given to each animal before the AMI-induction procedure to prevent the occurrence of malignant arrhythmia.

Under sterile conditions, the heart was exposed through mid-thoracotomy. The pericardium was gently removed and the mid-portion of left anterior descending (LAD) artery was ligated with 5–0 prolene suture just distal to the first diagonal branch. Regional myocardial ischemia is confirmed by typical changes in waveform on ECG monitor and the observation of rapid discoloration of myocardium from pink to gray over the anterior surface of left ventricle, together with the rapid development of akinesia and dilatation of at-risk area. AMI was confirmed by complete ECG following the procedure. After mid-LAD ligations, the mini-pigs were then categorized into AMI plus normal saline treatment (AMI-only) group (n=6) and AMI plus tacrolimus therapy (AMI-Tac) group (n=6). For the purpose of comparison at molecular-cellular levels, normal cardiac tissues were obtained from a group of six mini-pigs without receiving any treatment that served as the normal controls (N_C_).

The optimal drug dosage of tacrolimus has been demonstrated in our recent study to be 0.5 mg to achieve maximal efficacy and acceptable safety [26]. Thus, this dosage of tacrolimus (i.e., 0.5 mg for each animal) was utilized in the current study. By 30 minutes after LAD ligation, intra-coronary injections of physiological saline (3.0 mL) in AMI-only group and tacrolimus (0.5 mg in 2.5 mL physiological saline) in AMI-Tac group were given through the LAD beyond the point of ligation. The muscle and skin of the chest wall were then closed in layers. The animals were allowed to recover on the warming pad under close observation.

### Cardiovascular magnetic resonance imaging (MRI) analysis on days 2, 5 and 21 after AMI induction

The MRI protocol and procedure were according to our recent report [31]. Briefly, all animals in AMI group and AMI-Tac group underwent cardiovascular MRI studies on day 2, 5 and 21 after LAD ligation. All studies were performed by one investigator (with 15 years of experience in chest and cardiovascular MRIs) blinded to the treatment protocol using a 1.5T MR imager (Intera; Philips Medical Systems, Best, the Netherlands). After anesthesia, the mini pig was placed in a supine position with all four limbs fixed by Velcro strips. A phased array coil was wrapped around the chest. A cine MRI for assessment of LV volume and function was performed using a balanced steady-state free precession sequence with the following parameters: Repetition time msec/echo time msec, 3.7/1.8; section thickness, 10 mm; spacing, 0; flip angle, 70°; field of view, 26 × 26 cm; matrix size, 239 × 256; number of signals acquired, one; and, number of phases, 25. To measure the late gadolinium enhancement (LGE) MR imaging of the infarct area, 0.2 mmol/kg body weight (BW) gadopentetate dimeglumine (Magnevist, Bayer Schering Pharma, Berlin, Germany) was administered at 0.6 mL/s via the ear vein with a power injector (Spectris; Medrad, Indianola, PA) followed by a 5 mL saline flush. Twelve minutes post contrast injection, a 2D gradient-recalled-echo technique with gating & inversion recovery preparation was applied for late gadolinium enhancement (LGE) study with the inversion time (approximately 300–380 milliseconds) being optimized by myocardial nulling. LV functional parameters were determined by segmentation of the endocardial and epicardial contours of each slice during end-systole (ES) and end-diastole (ED) allowing for the evaluation of ED volume, ES volume, and ejection fraction (EF).

The mini pigs were sacrificed on day 21 after AMI induction for individual study. For the comparison of molecular-cellular biomarker changes among animals in the normal control, AMI without treatment, and AMI treated with tacrolimus groups, LV tissues from six other normal animals were utilized. These six animals did not receive cardiac MRI examination.

### Specimen collection, measurement of infarct area at basal, middle, and apical level of left ventricle

By day 21 after cardiac MRI examination, the twelve mini-pigs were sacrificed and the hearts were rapidly removed and immersed in cold saline and prepared for individual study. For immunohistofluorescence (IF) study, the heart tissue was rinsed with PBS, embedded in OCT compound (Tissue-Tek, Sakura, Netherlands) and snap-frozen in liquid nitrogen. Some heart tissue was also stored at −80°C for Western blot. For immunohistochemical (IHC) staining, heart tissue was fixed in 4% formaldehyde and embedded in paraffin.

Three cross-sections (1 cm in thickness) of the removed heart were obtained at identical basal, middle, and apical levels in each animal for measurement of myocardial infarct size in both placebo and cell-treated groups. To avoid the possibility of bias, the technicians who assessed the infarct area at basal, middle and apical levels were blinded to the therapeutic strategy. Each cross-section of heart tissue was then stained with trimethyl tetrazolium chloride (2% TTC) for infarct area analysis. Briefly, all heart sections were placed on a tray with a scaled vertical bar to which a digital camera was attached. The sections were photographed from directly above at a fixed height. The images obtained were then analyzed using Image Tool 3 (IT3) image analysis software (University of Texas, Health Science Center, San Antonio, UTHSCSA; Image Tool for Windows, Version 3.0, USA). The calculated infarct area (i.e., the average infarct area) was expressed as the percentage of infarct area (IA) compared with the total cross-sectional area of the left ventricle in each section for comparison [i.e. IA / (IA + non-IA) × 100%]. The rest of the cardiac tissue was then cut into pieces for specific studies.

For other molecular-cellular biological studies, the specimen of peri-IA was collected. To avoid incorrect tissue samplings of the peri-IA that would influence the outcome, the specimen of peri-IA (i.e., an area-at-risk) was collected at least 5 mm away from the edge of the infarct area.

The technicians/observers who performed imaging or molecular-cellular biological studies were blinded to the treatment protocol.

### Western blot analysis of left ventricular specimens

Equal amounts (30 μg) of protein extracts from the left ventricular myocardium were loaded and separated by SDS-PAGE using 8-10% acrylamide gradients. The membranes were incubated with the indicated primary antibodies against CD14 (1:1000, Abcam), CD68 (1:500, Abcam), Ly6G (1:1000, Abcam), connexin43 (Cx43) (1:1000, Chemicon), mitochondrial cytochrome C (1:2000, BD), tumor necrosis factor (TNF)-α (1: 1000, Cell Signaling), nuclear factor (NF)-κB (1:600, Abcam), interleukin (IL)-12 (1:500, Abcam), monocyte chemoattractant protein (MCP)-1 (1:500, Abcam), macrophage inflammatory protein (MIP)-1α (1:2000, Abcam), inducible nitric oxide synthase (iNOS) (1:250, Abcam), and γH2AX (1:1000, Cell signaling). Signals were detected with horseradish peroxidase (HRP)-conjugated goat anti-mouse, goat anti-rat, or goat anti-rabbit IgG.

Immunoreactive bands were visualized by enhanced chemiluminescence (ECL; Amersham Biosciences), which was then exposed to Biomax L film (Kodak). For quantification, ECL signals were digitized using Labwork software (UVP). For oxyblot protein analysis, a standard control was loaded on each gel.

### Immunohistochemical (IHC) staining

The procedure was based on our recent reports [25-27,32]. Briefly, paraffin sections (3 μm thick) were obtained from LV myocardium of each mini-pig. To block the action of endogenous peroxidase, the sections were initially incubated with 3% hydrogen peroxide, and then further processed using Beat Blocker Kit (Zymed Company, #50-300) with immersion in solutions A and B for 30 minutes and 10 minutes, respectively, at room temperature. Primary antibodies against CD14 (dilution 1/500; Spring Bioscience) and iNOS (dilution 1/200; Spring Bioscience) were then used, followed by application of SuperPicTure^TM^ Polymer Detection Kit (Zymed) for 10 minutes at room temperature. Finally, the sections were counterstained with hematoxylin. For negative control experiments, primary antibodies were omitted.

### Immunofluorescent (IF) imaging study

IF staining methodology for identification of phosphorylated variants of histone H2AX (γ-H2AX) in myocardium was utilized according to our recent reports [25-27,32]. The primary antibody [anti-γ-H2AX (Abcam)] and secondary antibody [Alex594-conjugated anti-mouse IgG (Molecular Probes)] were incubated for 30 minutes at room temperature. Irrelevant antibodies were used as control.

### Immunolabeling of connexin43 (Cx43) and quantitative image data analysis

Six serial sections of LV myocardium (three longitudinal and three transverse) were prepared at 4 μm thickness by Cryostat (Leica CM3050S) for Cx43 immunolabeling. To co-localize troponin I and Cx43 in the same sample, tissue sections were first incubated with a mixture of polyclonal anti-Cx43 (1:200) plus anti-troponin I (1:200) antibodies for 24 h at 4°C then incubated with anti-mouse FITC (1:200) and anti-rabbit Rhodamine (1:200) for 30 minutes at room temperature.

The integrated area (μm^2^) of Cx43 spots in the tissue sections was calculated using Image Tool 3 (IT3) image analysis software (University of Texas, Health Science Center, San Antonio, UTHSCSA; Image Tool for Windows, Version 3.0, USA) as described previously [25-27,32]. Three selected sections were quantified for each animal. Three randomly selected high-power fields (HPFs) (400 x) were analyzed in each section. After determining the number of pixels in each Cx43 spot per HPF, the numbers of pixels obtained from the three HPFs were summated. The procedure was repeated in two other sections for each animal. The mean pixel number per HPF for each animal was then determined by summating all pixel numbers and dividing by 9. The mean area of Cx43 per HPF was obtained using a conversion factor of 19.24 (1 μm^2^ represented 19.24 pixels).

### Statistical analysis

Quantitative data are expressed as means ± SD. Statistical analysis was adequately performed by ANOVA followed by Bonferroni multiple-comparison post hoc test. Statistical analysis was performed using SAS statistical software for Windows version 8.2 (SAS institute, Cary, NC). A probability value <0.05 was considered statistically significant.

## Results

### Time courses of cardiac MRI findings after AMI induction

**Figure 1:**
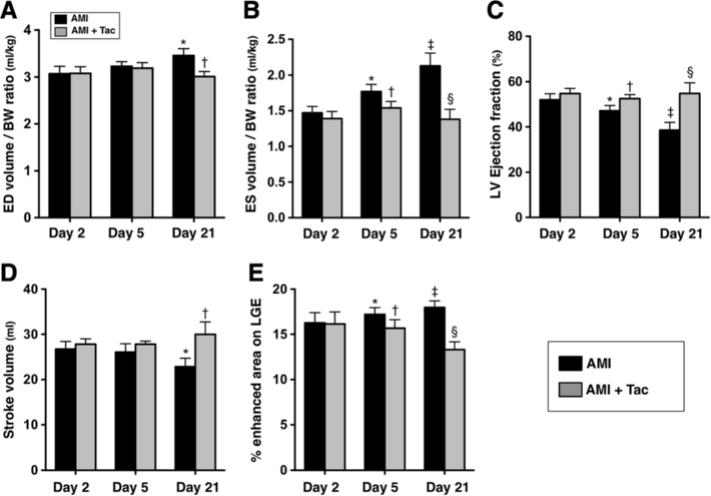
**Cardiac magnetic resonance imaging (MRI) findings. A)** The ratio of end diastolic (ED) volume to body weight (BW) by days 2, 5 and 21 after acute myocardial infarction (AMI) induction. By day 21, * vs. †, p<0.005. **B)** The ratio of end systolic (ES) volume to BW by days 2, 5 and 21 after AMI induction. By day 5, * vs. †, p<0.01. By day 21, ‡ vs. §, p<0.01. **C)** Left ventricular (LV) ejection fraction by days 2, 5 and 21 after AMI induction. By day 5, * vs. †, p<0.01. By day 21, ‡ vs. §, p<0.01. **D)** Stroke volume by days 2, 5 and 21 after AMI induction. By day 21, * vs. †, p<0.01. **E)** % of enhanced area on late gadolinium enhancement (LGE) by days 2, 5 and 21 after AMI induction. By day 5, * vs. †, p<0.01. By day 21, ‡ vs. §, p<0.01. Tac = tacrolimus. (n=6 for each group).

The ratio (R) of end-diastolic volume (EDV) to the body weight (BW) (R_EDV/BW_) (ml/kg) did not differ between AMI group and AMI-Tac group at day 2 and day 5 after AMI induction (Figure 1A). However, the R_EDV/BW_ was significantly higher in AMI group than in AMI-Tac group by day 21 after AMI induction. Additionally, the ratio of end-systolic volume (ESV) to BW (R_ESV/BW_) was similar between the AMI group and AMI-Tac group by day 2 after AMI induction. However, this parameter was significantly higher in AMI group than in AMI-Tac group by day 5 and day 21 after AMI induction (Figure 1B). These findings suggest that tacrolimus therapy significantly inhibited LV remodeling after AMI.

The LVEF did not differ between the AMI group and AMI-Tac group by day 2 after AMI induction. However, the LVEF was significantly lower in AMI group than that in AMI-Tac group by day 5 (Figure 1C). Moreover, by day 21, this parameter was significantly lower in the AMI group compared with that in AMI-Tac group. These findings imply that tacrolimus therapy significantly improved LV function after AMI.

The stroke volume did not differ between the AMI group and AMI-Tac group by days 2 and 5 after AMI induction (Figure 1D). However, the stroke volume was significantly increased in AMI-Tac group than in AMI group by day 21. These findings suggest once more tacrolimus therapy significantly preserved LV function after AMI.

The LGE of the infarct area was similar between the AMI group and AMI-Tac group by day 2 after AMI induction. However, the transmural LGE was significantly reduced by day 5 and more significantly reduced by day 21 in AMI-Tac group than in AMI group. These findings implicate that tacrolimus treatment significantly reduced tranasmural infarct size and prevented myocardium from death.

### Assessment the infarct area of LV myocardium and thickness of LV wall

**Figure 2:**
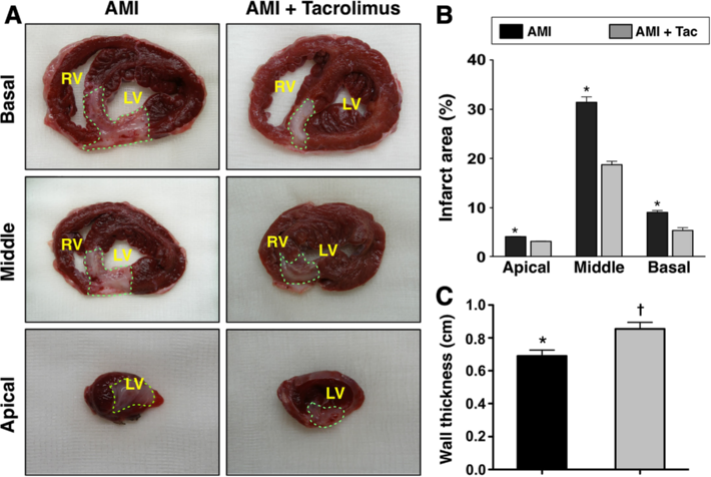
**Histopathological findings of infarct area by day 21 after AMI. A)** Illustrations of trimethyl tetrazolium chloride (TTC) staining of the left ventricular (LV) infarct area at basal, middle and apical areas by day 21 after AMI induction. **B)** Statistical analysis showed significantly lower infarct area at basal, middle and apical levels of left ventricle in AMI + Tac group than in AMI group (all p values <0.05). **C)** Quantification of wall thickness in infarct area. * vs. †, p<0.05. Tac = tacrolimus. (n=6 for each group).

Figure 2 shows the results of TTC staining by day 21 following AMI. As expected, the IA was notably larger in AMI group than that in AMI-Tac group at basal, middle, and apical levels (Figure 2A). Accordingly, the quantification of the IA showed significantly larger area of infarction at all three levels in AMI group than that in AMI-Tac group (Figure 2B). By contrast, summation of the wall thickness at all three levels demonstrated significantly thinner LV wall in the AMI group as compared with that in the AMI-Tac group (Figure 2C). These findings may partially explain the notably higher LVEF and remarkably reduced LV remodeling in AMI animals with tacrolimus treatment compared to those without.

### Measurement of Innate immune response in the circulation and in IA and peri-IA by Day 21 after AMI induction

**Figure 3:**
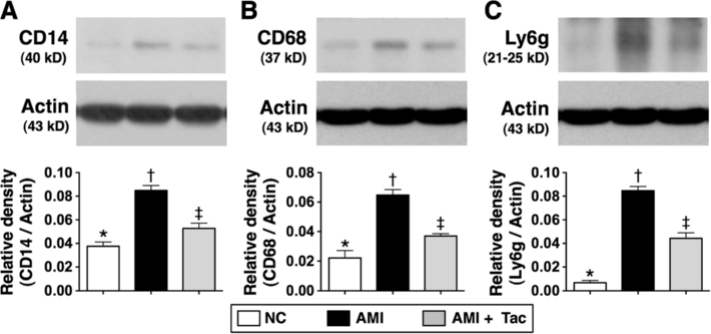
**Protein extraction of innate immune cells in the circulation by day 21 after AMI induction. A)** Protein expression of CD14 extracted from peripheral-blood mononuclear cells. * vs. other bars with different symbols, p<0.005. **B)** Protein expression of CD68 extracted from peripheral-blood macrophages. * vs. other bars with different symbols, p<0.005. **C)** Protein expression of Ly6G extract from circulating neutrophils. * vs. other bars with different symbols, p<0.001. All statistical analyses using one-way ANOVA, followed by Bonferroni multiple comparison post hoc test. Symbols (*, †, ‡) indicate significance (at 0.05 level). NC = normal control. AMI = acute myocardial infarction; Tac = tacrolimus. (n=6 for each group).

The protein expressions of CD14 (extracted from peripheral-blood mononuclear cells), CD68 (extracted from peripheral-blood macrophages), and Ly6G (extract from circulating neutrophils), three indices of innate immune response, were significantly higher in AMI group than those in AMI-Tac group and normal controls (N_C_), and significantly higher in AMI-Tac group than those in N_C_ (Figure 3).

**Figure 4:**
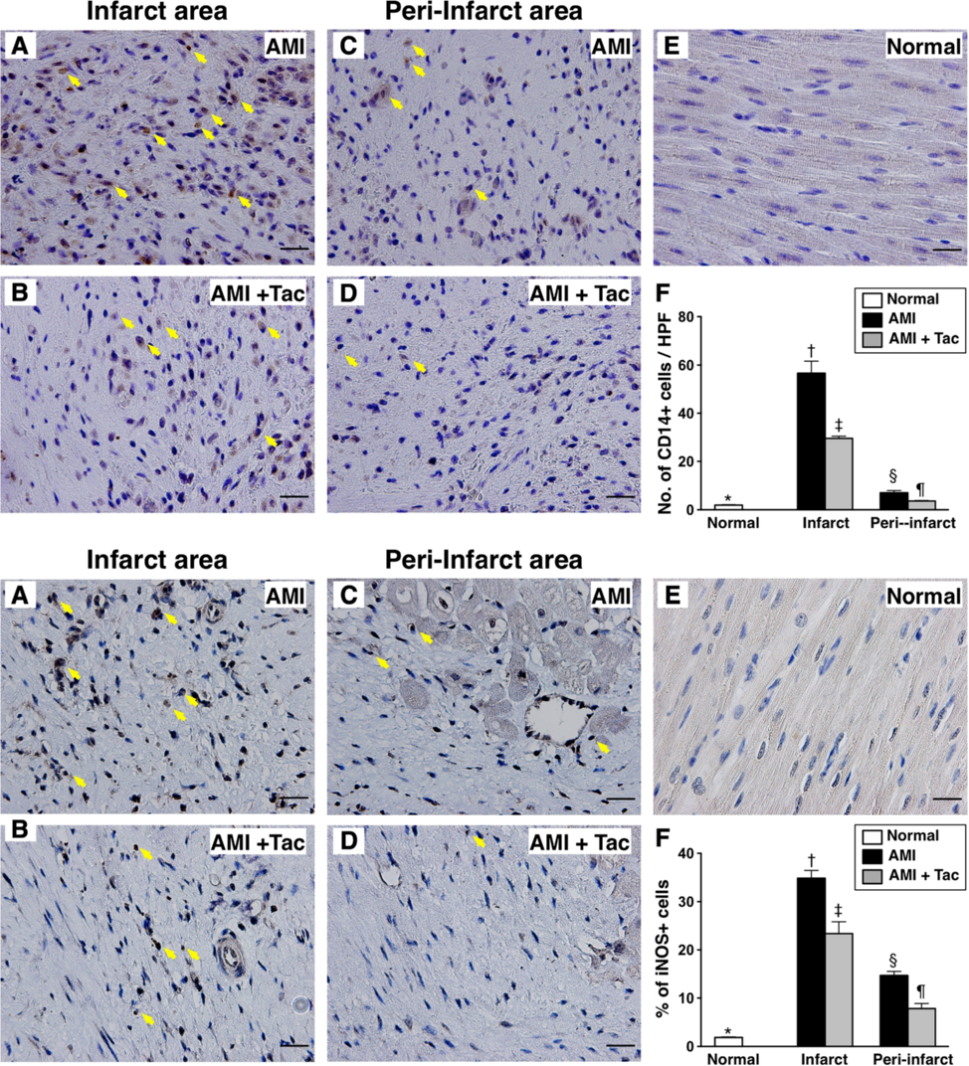
**Immunohistochemical (IHC) staining of the innate inflammatory cells in infarct area (IA) and peri-IA at day 21 after AMI induction.** Upper panel: **A to E)** Illustration of IHC staining of CD14+ cells (yellow arrows) in infarct area (IA) and peri-IA of left ventricle. **F)** Quantification of number of CD14+ cells. At IA, * vs. other bars with different symbols, p<0.0001. At peri-IA, * vs. other bars with different symbols, p<0.01. (n=6 for each group). Lower panel: **A to E)** Illustration of IHC staining of inducible nitric oxide synthase (iNOS) + cells (yellow arrows) in IA and peri-IA of left ventricle. **F)** Quantification of iNOS+ cells. At IA, * vs. other bars with different symbols, p<0.0001. At peri-IA, * vs. other bars with different symbols, p<0.001. All statistical analyses using one-way ANOVA, followed by Bonferroni multiple comparison post hoc test. Symbols (*, †, ‡ or *, §, ¶) indicate significance (at 0.05 level). HPF = high-power field; AMI = acute myocardial infarction; Tac = tacrolimus. (n=6 for each group).

IHC staining showed that the numbers of CD14+ cells (Figure 4, upper panel) and inducible NO synthase (iNOS)+ (i.e., the innate immune signaling cascade) (Figure 4, lower panel), two indicators of innate immune response, in both IA and peri-IA were significantly higher in AMI group than those in AMI-Tac group and N_C_, and significantly higher in AMI-Tac group than those in N_C_.

### Determination of protein expressions of innate inflammatory reaction in IA and Peri-IA by day 21 after AMI induction

**Figure 5:**
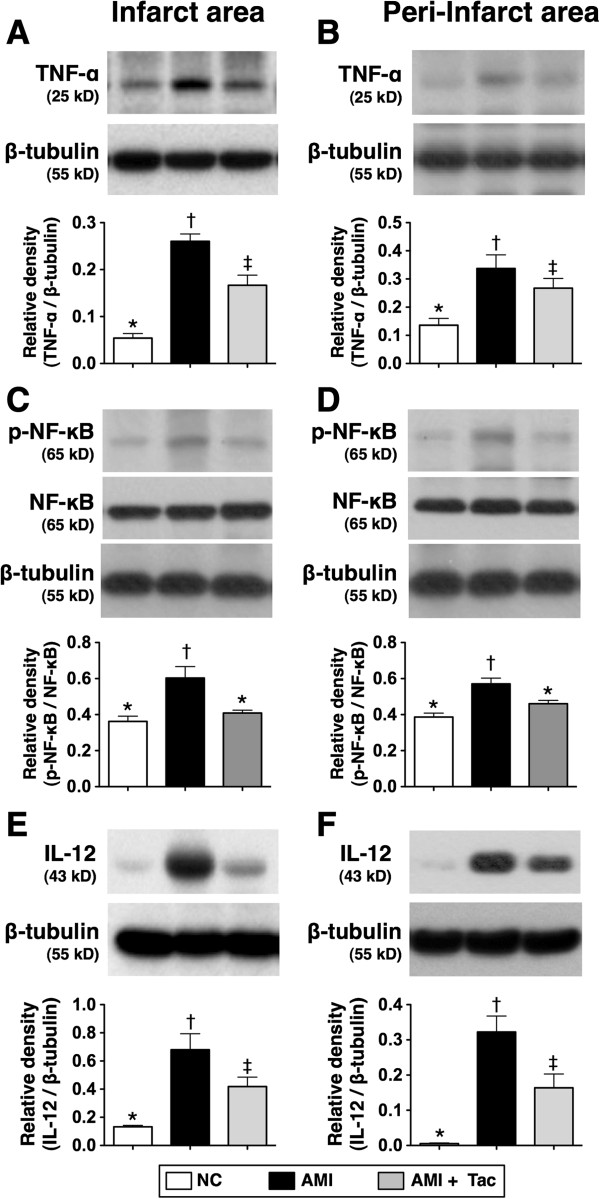
**Protein expressions of innate inflammatory biomarkers of TNF-α, NF-κB, and IL-12 by day 21 after AMI induction. A)** The protein expressions tumor necrotic factor (TNF)-α in infarct area (IA). * vs. other bars with different symbols, p<0.001. **B)** The protein expression of TNF-α in per-IA. * vs. other bars with different symbols, p<0.01. **C)** The ratio of protein expression of phosphorylated (p) nuclear factor (NF)-κB/total NF-κB in IA. * vs. other bars with different symbols, p<0.01. **D)** The ratio of protein expression of p-NF-κB/total NF-κB in peri-IA. * vs. other bars with different symbols, p<0.05. **E)** Protein expression of interleukin (IL)-12 in IA. * vs. other bars with different symbols, p<0.001. **F)** Protein expression of IL-12 in peri-IA. * vs. other bars with different symbols, p<0.0001. All statistical analyses using one-way ANOVA, followed by Bonferroni multiple comparison post hoc test. Symbols (*, †, ‡) indicate significance (at 0.05 level). NC = normal control; AMI = acute myocardial infarction; Tac = tacrolimus. (n=6 for each group).

**Figure 6:**
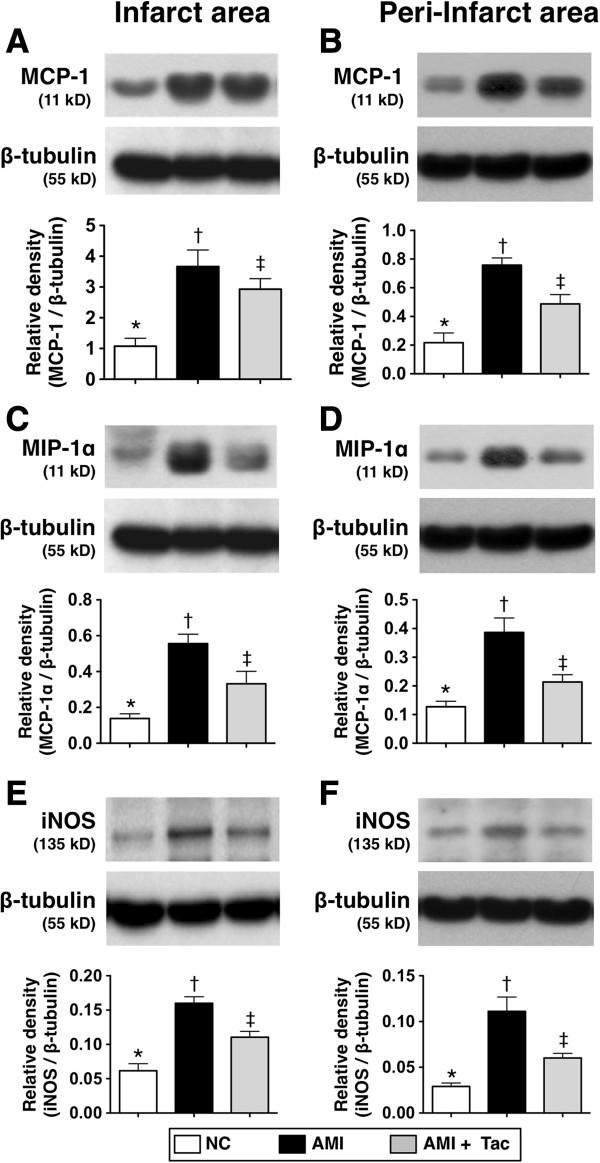
**Protein expressions of innate inflammatory biomarkers of MCP-1, MIP-1, and iNOS by day 21 after AMI induction. A)** The protein expressions monocyte chemoattractant protein (MCP)-1 in infarct area (IA). * vs. other bars with different symbols, p<0.01. **B)** The protein expression of MCP-1 in peri-IA. * vs. other bars with different symbols, p<0.01. **C)** The protein expression of macrophage inflammatory protein (MIP)-1 in IA. * vs. other bars with different symbols, p<0.01. **D)** The protein expression of MIP-1 in peri-IA. * vs. other bars with different symbols, p<0.01. **E)** The protein expression of inducible nitric oxide synthase (iNOS) in IA. * vs. other bars with different symbols, p<0.01. **F)** The protein expression of iNOS in peri-IA. * vs. other bars with different symbols, p<0.01. All statistical analyses using one-way ANOVA, followed by Bonferroni multiple comparison post hoc test. Symbols (*, †, ‡) indicate significance (at 0.05 level). NC = normal control; AMI = acute myocardial infarction; Tac = tacrolimus. (n=6 for each group).

The protein expressions of TNF-α, IL-12 (Figure 5), MCP-1, MIP-1α, and iNOS (Figure 6), six indices of innate inflammatory response in IA and peri-IA, were significantly higher in AMI group than those in AMI-Tac group and N_C_, and significantly higher in AMI-Tac group than those in N_C_. Additionally, the ratio of phosphorylated- NF-κB/total NF-κB was significantly higher in AMI group than in AMI-Tac group and N_C_, but it showed no difference between AMI-Tac group and N_C_.

### Immunofluorescent microscopic findings of myocardial integrity biomarkers in peri-IA and non-IA by day 21 after AMI induction

**Figure 7:**
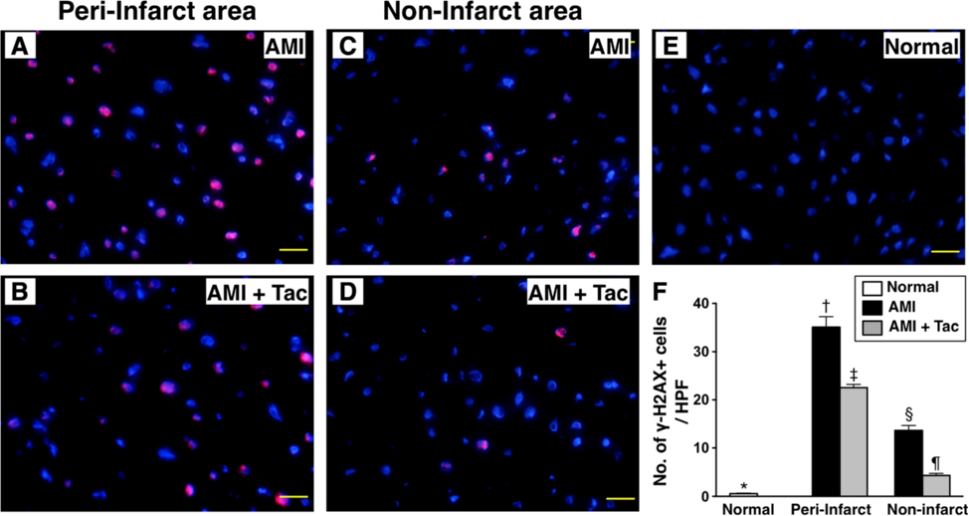
**Immunofluoroscopic findings of γ-H2AX+ cells in peri-infarct area (IA) and non-IA by day 21 after AMI induction. A to E)** The illustration of immunofluorescent (IF) stain of γ-H2AX+ cells in peri-IA and non-IA. **F)** Quantification of number of γ-H2AX+ cells in peri-IA and non-IA. At peri-IA, * vs. other bars with different symbols, p<0.0001. At non-IA, * vs. other bars with different symbols, p<0.001. All statistical analyses using one-way ANOVA, followed by Bonferroni multiple comparison post hoc test. Symbols (*, †, ‡ or *, §, ¶) indicate significance (at 0.05 level). HPF = high-power field; NC = normal control; AMI = acute myocardial infarction; Tac = tacrolimus. (n=6 for each group).

**Figure 8:**
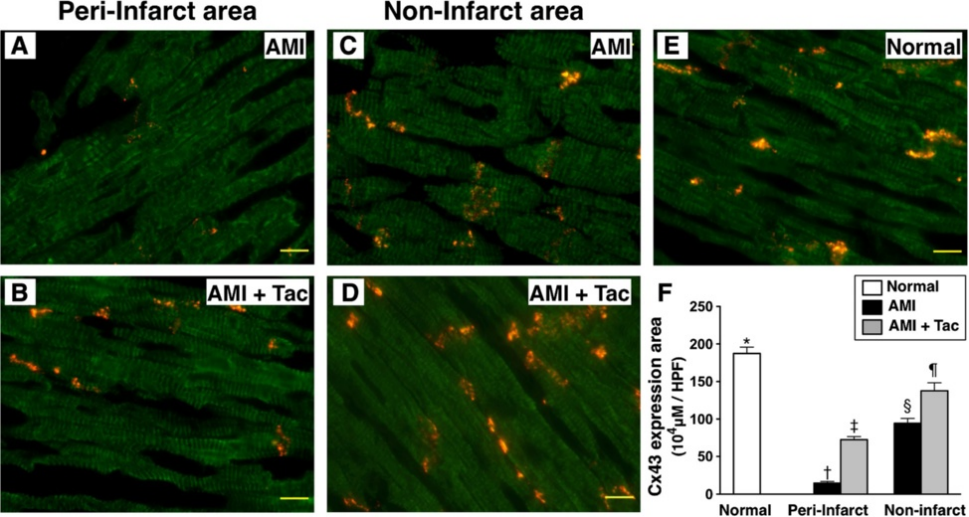
**Immunofluoroscopic findings of connexin43 expression in peri-infarct area (IA) and non-IA by day 21 after AMI. A to E)** The illustration of immunofluorescent (IF) stain of connexin43 (Cx43) expression in peri-IA and non-IA. **F)** Quantification of CX43 distribution (area) in per-IA and non-IA. At peri-IA, * vs. other bars with different symbols, p<0.0001. At non-IA, * vs. other bars with different symbols, p<0.001. All statistical analyses using one-way ANOVA, followed by Bonferroni multiple comparison post hoc test. Symbols (*, †, ‡ or *, §, ¶) indicate significance (at 0.05 level). HPF = high-power field; AMI = acute myocardial infarction; Tac = tacrolimus. (n=6 for each group).

IF staining demonstrated that the number of γ-H2AX+ cells, an index of DNA damage in peri-IA and non-IA, was significantly higher in AMI group than that in AMI-Tac group and N_C_, and significantly higher in AMI-Tac group than that in N_C_ (Figure 7). On the other hand, IF also revealed that the Cx43-expression area, an indicator of integrity of gap junction between cardiomyocytes in peri-IA and non-IA, was significantly reduced in AMI group than in AMI-Tac group and N_C_, and significantly reduced in AMI-Tac group than that in N_C_ (Figure 8). These findings support that tacrolimus treatment protected cardiomyocytes from damaged after AMI induction.

### Examination of protein expressions of myocardial integrity biomarkers in IA and peri-IA by day 21 after AMI induction

**Figure 9:**
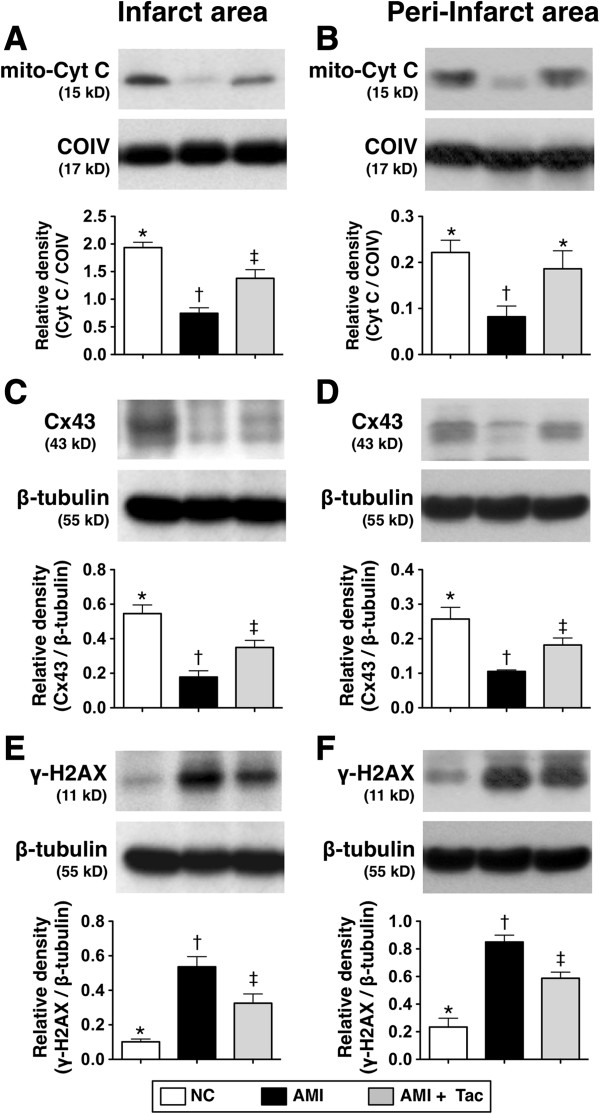
**Protein expressions of myocardial integrity biomarkers of mitochondrial cytochrome C, connexin43 and γ-H2AX in infarct area (IA) and peri-IA by day 21 after AMI induction. A)** protein expression of mitochondrial cytochrome C in IA. * vs. other bars with different symbols, p<0.005. **B)** protein expression of mitochondrial cytochrome C in peri-IA. * vs. other bars with different symbols, p<0.01. **C)** protein expression of connexin43 (Cx43) in IA. * vs. other bars with different symbols, p<0.01. **D)** protein expression of Cx43 in peri-IA. * vs. other bars with different symbols, p<0.01. **E)** Protein expression of γ-H2AX in IA. * vs. other bars with different symbols, p<0.001. **F)** Protein expression of γ-H2AX in peri-IA. * vs. other bars with different symbols, p<0.001. All statistical analyses using one-way ANOVA, followed by Bonferroni multiple comparison post hoc test. Symbols (*, †, ‡) indicate significance (at 0.05 level). NC = normal control; AMI = acute myocardial infarction; Tac = tacrolimus. (n=6 for each group).

The protein expression of mitochondrial cytochrome-C, and index of mitochondrial integrity and energy preservation in IA and peri-IA, was significantly lower in the untreated AMI group than that in AMI-Tac group and N_C_, and significantly lower in AMI-Tac group than that in N_C_ in IA, but it showed no difference between the latter two groups in peri-IA (Figure 9A and B). Additionally, the protein expression of Cx43 in IA and peri-IA was significantly lower in AMI group than that in AMI-Tac group and N_C_, and significantly lower in AMI-Tac group than that in N_C_ (Figure 9C and D). However, the protein expression of γ-H2AX in IA and peri-IA showed an opposite pattern compared to that of Cx43 among the three groups (Figure 9E and F).

**Figure 10:**
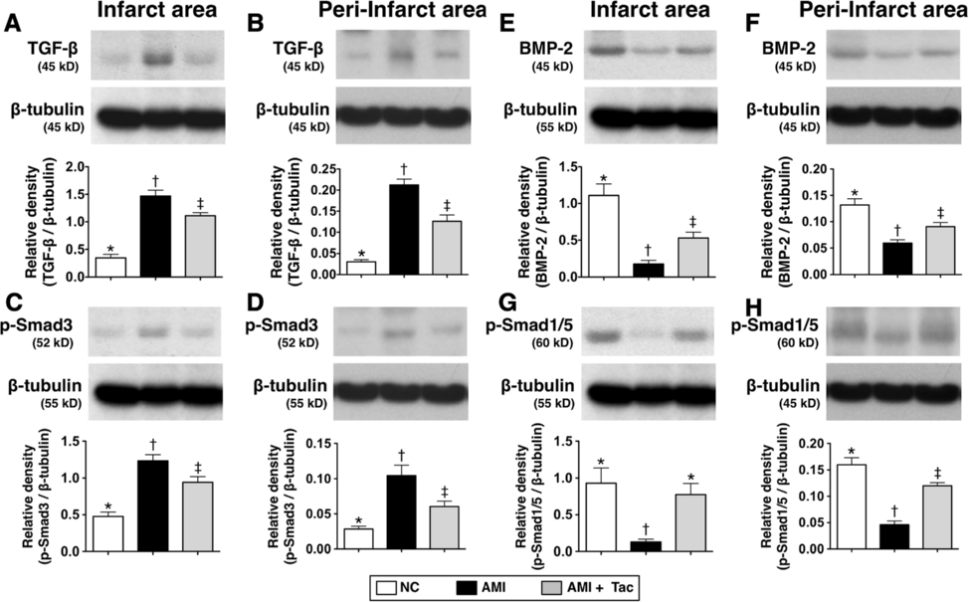
**Protein expressions of fibrotic and anti-fibrotic markers in infarct area (IA) and peri-IA by day 21 after AMI. A)** The protein expression of transforming growth factor (TGF)-β in IA. * vs. other bars with different symbols, p<0.001. **B)** The protein expression of TGF-β in peri-IA. * vs. other bars with different symbols, p<0.001. **C)** The protein expression of phosphorylated (p)-Smad3 in IA. * vs. other bars with different symbols, p<0.01. **D)** The protein expression of p-Smad3 in peri-IA. * vs. other bars with different symbols, p<0.01. **E)** The protein expression of bone morphogenic protein (BMP)-2 in IA. * vs. other bars with different symbols, p<0.001. **F)** The protein expression of BMP-2 in peri-IA. * vs. other bars with different symbols, p<0.01. **G)** The protein expression of p-Smad1/5 in IA. * vs. other bars with different symbols, p<0.001. **H)** The protein expression of p-Smad1/5 in peri-IA. * vs. other bars with different symbols, p<0.001. All statistical analyses using one-way ANOVA, followed by Bonferroni multiple comparison post hoc test. Symbols (*, †, ‡) indicate significance (at 0.05 level). NC = normal control; AMI = acute myocardial infarction; Tac = tacrolimus. (n=6 for each group).

The protein expressions of TGF-β and Smad3, two fibrotic biomarkers in IA and peri-IA, were significantly higher in animals with untreated AMI than that in the AMI-Tac group and N_C_, and significantly higher in the AMI-Tac group than that in N_C_ (Figure 10A to D). Conversely, the protein expressions of BMP-2 and Smad1/5, two anti-fibrotic indices in IA and peri-IA, showed an opposite pattern compared to that of fibrotic biomarkers in the three groups (Figure 10E to H).

## Discussion

This study, which investigated the role of innate immune response and the impact of tacrolimus therapy in mini-pig AMI model, yielded several striking implications. First, not only was the innate immune system in circulation enhanced in circulation after AMI induction, but it was also significantly activated in the myocardium. Second, the expressions of myocardial damage biomarkers were markedly augmented, whereas those of myocardial integrity were remarkably reduced after AMI. Third, myocardial IA and LV remodeling were notably suppressed, whereas the LVEF was significantly preserved after tacrolimus therapy. Accordingly, the results of this study highlight the therapeutic potential of tacrolimus in the setting of AMI.

### Distinctive therapeutic potential of tacrolimus against AMI --- changes in IA and cardiac MRI assessment of LV function and remodelling

The most important finding in the present study is the remarkable preservation of LVEF after AMI induction in animals with tacrolimus treatment compared to those without. Moreover, another distinctive finding in the present study is the reduction in AMI-related LV remodeling after tacrolimus treatment.

The pathological findings of myocardial infarction after LAD ligation confirmed the successful establishment of the animal model. Histologically, IA at apical, middle, and basal levels of left ventricle was markedly increased after induction of myocardial infarction but was substantially reduced following tacrolimus treatment. Fascinatingly, although it is conceivable that the low tacrolimus dosage for local cardiac administration probably caused a very low circulating drug level compared to its use as immunosuppressant, it remarkably reduced infarct size, suppressed LV remodeling, and preserved cardiac function. Such distinct advantage highlights the therapeutic potential of tacrolimus as a safe and efficient cardiac protective agent, instead of its routine use as a systemic immunosuppressant.

Undoubtedly, cardiac MRI is more accurately than M-mode echocardiography for measuring the left ventricular function, especially after AMI. Using echocardiography, our previous study showed that as compared to AMI group, AMI + tacrolimus significantly improved LVEF and attenuated LV chamber size by day 14 after AMI induction [26]. In the present study, we utilized cardiac MRI to measure the serial changes of LVEF, stroke volume and LV dimension and the results exhibited that tacrolimus therapy significantly improved heart function and inhibited LV remodeling. In this way, the MRI findings of the present study support the echocardiography findings of our previous study [26]. Of particularly important finding was that the cardiac MRI rather than the echocardiography provided the useful information of transmural LGE in the infarct area that could accurately assess that percentage of the infarct wall and the survival of myocardium after AMI induction with and without tacrolimus therapy. In this way, the cardiac MRI is superior to echocardiography for identifying the myocardial integrity after AMI.

### Circulating inflammatory reaction after AMI --- innate immune signaling

Undoubtedly, AMI elicits tremendous local and systemic inflammatory responses [21-25]. One important finding in the present study was that the CD14, CD68, and Ly6G, typical and critical cell expressing innate markers, is the remarkably in circulation after AMI. This finding supports that an augmented innate immune response frequently occurs in the circulation after AMI. Our finding not supports those from previous studies [21-25] but also highlights that the innate immune system is throughout activated in circulation. Of importance is that these biomarkers of innate immunity were remarkably attenuated after tacrolimus treatment. The possible mechanism underlying the observation would be the inhibition of myocardial inflammatory reaction that, in turn, suppressed the expression of inflammatory mediators in the circulation.

### Local inflammatory reaction in myocardium after AMI -- innate immune signaling and myocardial damage

Propagation of inflammatory reaction has been well recognized as a significant contributor to myocardial damage after AMI [22-27]. However, the extent of involvement of the innate immune system in regulating the inflammatory reaction after AMI has not been fully investigated [25,27]. An essential finding in the present study is that not only the protein (i.e., TNF-α, NF-κB, IL-12, MCP-1, MIP-1α, and iNOS), but also the cellular (CD14+ and iNOS+ cells) expressions of innate immune reactions were found to be involved in AMI-induced inflammatory process in myocardium. Thus, our findings implicate that the inflammation and immune response is actually the coin of both side, very close related to each other after AMI induction. Additionally, our findings once gain support that the innate immune system is not only throughout activated in circulation but it also entirely augmented in infarction area after AMI. Moreover, these findings, in addition to reinforcing those from previous studies [22-27], could at least in part explain the aggravated pathological cardiac changes in animals without treatment. Of particularly importance is the finding of significantly reduced IA animals after tacrolimus treatment. The results could partially explain the notably preserved LVEF and alleviated LV remodeling in the tacrolimus-treated animals.

### Effects of tacrolimus on myocardial damage and integrity

Previous studies have clearly demonstrated that the expressions of myocardial damage and remodeling biomarkers (i.e., TGF-β, Smad3, MHC-β, cytosolic cytochrome C) were markedly enhanced after AMI [21,25-27,32]. By contrast, the expressions of myocardial integrity biomarkers (i.e., BMP-2, Smad1/5, Cx43, MHC-α, mitochondrial cytochrome C) were remarkably decreased [21,25-27,32]. Of importance is that link between the increased expressions of myocardial damage parameters that stand for the propagation of inflammatory reactions and the deterioration in cardiac function. In this way, our findings are consistent with those of previous studies [21,25-27,32]. One important finding in the present study is that notably attenuated expressions of myocardial damage biomarkers in AMI animals after tacrolimus treatment. These findings again partially explain the preserved LVEF in tacrolimus-treated animals after AMI. Our finding, therefore, not only explains the remarkably suppressed LV remodeling after tacrolimus treatment, but also supports the use of this agent as a potential adjunctive therapy for patients with AMI having just received primary PCI.

### Why we utilized the same animal model in three consecutive studies to determining the therapeutic effect of tacrolimus on STEMI?

The same animal model and identical strategic treatment had been performed by our two previous studies [26,27]. In our two previous studies [26,27], we have measured the inflammatory and apoptotic biomarkers, the generations of oxidative stress and antioxidants, and MAPK signaling pathway, respectively. In the present study, we focused on assessing the innate immune response. The results of the present study provided critical complementary information that associate with the data from our previous studies [26,27] can, therefore, highlight a more completely history of molecular-cellular perturbations, inflammatory reaction and the immune response in setting of AMI, as well as the potentially accessorily therapeutic impact of tacrolimus for STEMI patients in the near future.

### Study limitations

**Figure 11:**
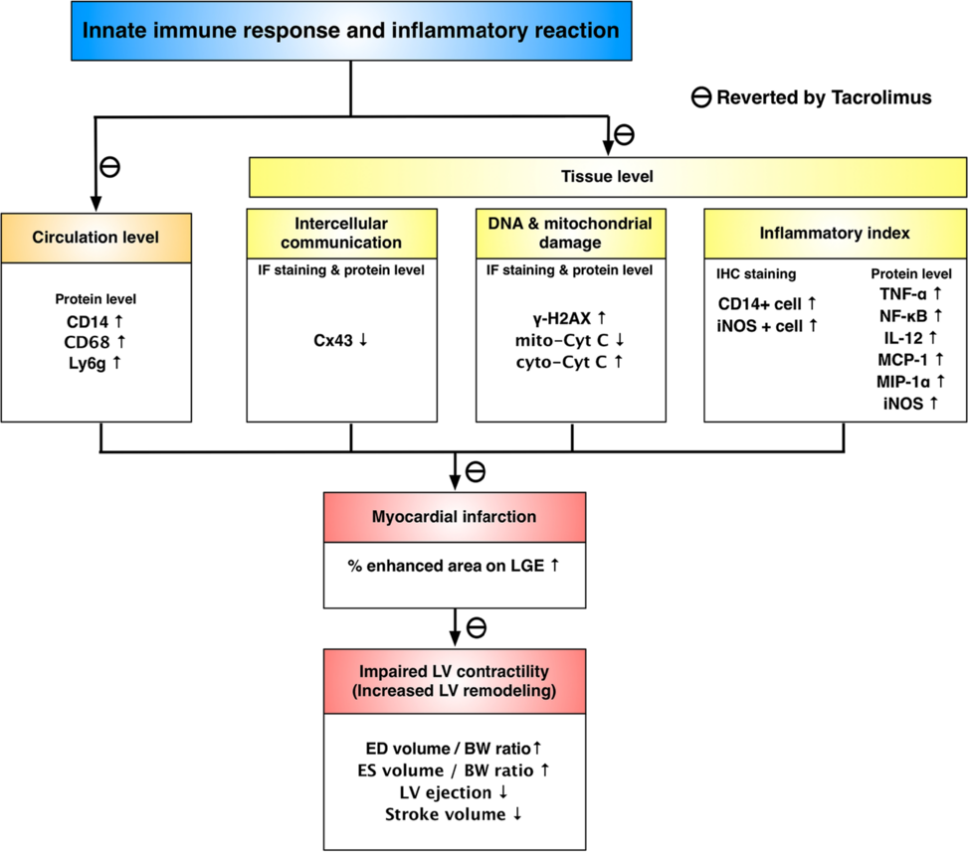
**Proposed mechanisms underlying the effects of tacrolimus therapy on preservation of heart function in a min-pig AMI model based on the findings of the present study.** IF = immunofluorescent; IHC = immunohistochemical; Cx43 = connexin43; Mito-Cyt C = mitochondrial cytochrome C; Cyto-Cyt C = cytosolic cytochrome C; LGE = late gadolinium enhancement; LV = left ventricular; ED = end diastolic; ES = end stroke; BW = body weight.

This study has limitations. First, without examination of adaptive immune system, the extent of participation of this immune system in regulating the inflammatory process after the occurrence of AMI remains to be elucidated. Second, since the current study did not investigate mitochondrial dysfunction [i.e., the opening of the mitochondrial permeability-transition (MPT) pore after AMI], whether heart function was preserved through regulation of the MPT pore in damaged myocardium after tacrolimus therapy in AMI animals is also unclear. Third, the cardiac MRI measurement was done on days 2, 5 and 21 after AMI induction rather than the basal values at day 0 prior to the induction of AMI on the AMI and AMI + Tac groups. Thus, the acute effect of AMI on cardiac functions did not provide from the results of the present study. Finally, although extensive work had been done in the current study, the exactly underlying mechanisms of the innate immunity has played an important role in the pathogenesis of AMI is still not entirely investigated and that the mechanistic basis involved in the improvement of heart function after tacrolimus therapy has not been fully clarified. The proposed mechanisms have been summarized in Figure 11 based on our findings.

## Conclusion

The innate immune system was significantly and extensively upregulated after AMI in a swine model. Tacrolimus therapy effectively suppressed the inappropriate innate immune response and LV remodeling, thereby significantly preserving LVEF after AMI in this experimental setting.

## Competing interests

The authors declare that they have no competing interests.

## Authors’ contributions

JJS, PHS, SL, SFK, and HKY designed the experiment, performed animal experiments, and drafted the manuscript. FYL and YYZ performed the animal experiments. HTC, YYZ, YCC, SC, and YLC were responsible for the laboratory assay and troubleshooting. HWC, SFK, and HKY participated in refinement of experiment protocol and coordination and helped in drafting the manuscript. All authors have read and approved the final manuscript.
